# Reactivity of Heteropolytungstate and Heteropolymolybdate Metal Transition Salts in the Synthesis of Dimethyl Carbonate from Methanol and CO_2_

**DOI:** 10.3390/ijms11072770

**Published:** 2010-07-23

**Authors:** Ahmed Aouissi, Salem S. Al-Deyab, Ahmad Al-Owais, Amro Al-Amro

**Affiliations:** 1 Department of Chemistry, King Saud University, P. O. Box 2455, Riyadh 11451, Saudi Arabia; 2 Petrochemical Research Chair, Department of Chemistry, King Saud University, P. O. Box 2455, Riyadh 11451, Saudi Arabia

**Keywords:** heteropoly compounds, dimethyl carbonate, Keggin structure, carbon dioxide

## Abstract

A series of Keggin-type heteropoly compounds (HPC) having different countercations (Co, Fe) and different addenda atoms (W, Mo) were synthesized and characterized by means of Fourier-Transform Infrared Spectrometer (FT-IR) and X-ray powder diffraction (XRD). The catalytic properties of the prepared catalysts for the dimethyl carbonate (DMC) synthesis from CO_2_ and CH_3_OH were investigated. The experimental results showed that the catalytic activity is significantly influenced by the type of the countercation and addenda atoms transition metal. Among the catalysts examined, Co_1.5_PW_12_O_40_ is the most active for the DMC synthesis, owing to the synergetic effect between Co and W. Investigating the effect of the support showed that the least acidic one (Al_2_O_3_) enhanced the conversion but decreased the DMC selectivity in favor of that of methyl formate (MF), while that of dimethoxy methane remained stable.

## 1. Introduction

The limited fossil fuel resources and the problems of global warming caused by an increase of atmospheric carbon dioxide concentrations have stimulated research in the utilization of CO_2_. One of the remedies for these problems is the development of processes that could economically convert CO_2_ into fuels or useful chemicals. The efficient transformation of carbon dioxide into useful chemical compounds is very attractive because it is a potentially inexpensive and abundant C1 building block, and it is environmentally benign (nontoxic, noncorrosive, and nonflammable) [1,2]. In recent years, various chemical processes have been tried to convert CO_2_ into valuable chemical compounds [3–5]. An increased use of CO_2_ would only be possible if the relatively inert CO_2_ molecule could be activated. Among the chemical compounds that can be obtained by using CO_2_, dimethyl carbonate (DMC) is considered as one of the most important. It can be synthesized by reaction of methanol and CO_2_. Therefore, it is required to find highly reactive metal catalysts that can activate the relatively inert CO_2_ molecule. Various catalysts have been reported to catalyze this reaction [6–16]. It is worth noting that DMC is an important raw material in organic synthesis and has drawn much attention of researchers. It has been paid more and more attention due to its low toxicity and wide applications [17]. It can be used as an environment-friendly intermediate and starting material for organic synthesis via carbonylation and methylation, replacing poisonous phosgene and dimethyl sulfate [18]. It is also considered an option for meeting the oxygenate specifications on gasoline [17]. Although the direct synthesis of DMC from methanol and CO_2_ is a promising route, nevertheless, DMC yield is relatively low due to the fact that CO_2_ is highly thermodynamically stable and kinetically inert and due to the deactivation of catalysts by *in situ* produced water. The problems associated with liquid phase processes can be solved by the development of an effective heterogeneous catalyst that can facilitate the CO_2_ activation. For that purpose, various catalysts have been tested in the activation of CO_2_ [19–22]. Among them, cobalt-based and iron-based catalysts have been reported effective for the activation of CO_2_ [19,23,24]. In this work, a series of cobalt and iron heteropolyoxometalate catalysts were prepared and tested for the direct synthesis of DMC in liquid-phase. The influence of the cobalt and iron as a countercation in the Keggin type heteropolytungstate and hetropolymolybdate was investigated. It is well known that the acidity and redox properties of 12-heteropoly compounds depend on both the constituent elements of polyanions and countercations.

## 2. Results and Discussion

### 2.1. Characterization of Catalysts

**Figure 1.**
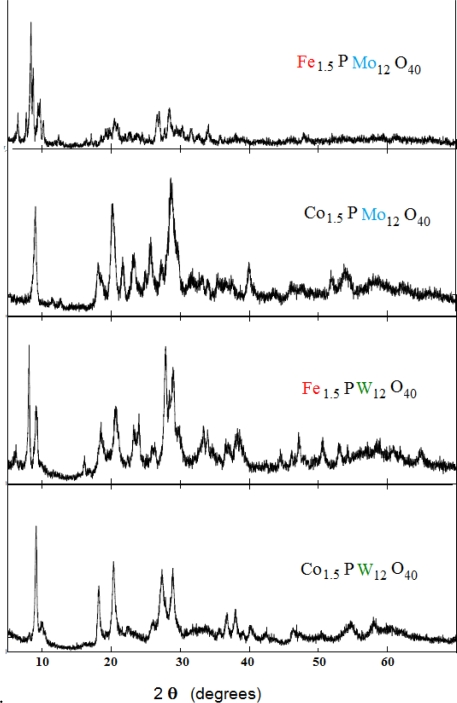
X-ray powder diffraction (XRD) patterns of PW_12_O_40_ and PMo_12_O_40_ having cobalt and iron as countercations.

IR spectra of the heteropoly compounds are shown in Table 1. The IR spectra have been assigned according to Ref [25]. The main characteristic features of the Keggin structure are observed at 1080−1060 cm^−1^, 990−960 cm^−1^, 900−870 cm^−1^, and 810−760 cm^−1^ assigned to the stretching vibration ν_as_ (P-O_a_), ν_as_ (M-O_d_), ν_as_ (M-O_b_-M), and ν_as_ (M-O_c_-M), respectively (M = W or Mo). The result of X-ray powder diffraction (XRD) of the product is shown in Figure 1. In each of the four ranges of 2θ, 7°–10°, 16°–23°, 25°–30°, and 31°–38°, the compound shows a characteristic peak of heteropolyanions (HPA) having Keggin structure [26–28]. Therefore, the presence of the primary Keggin structure in the synthesized phases was confirmed by FT-IR and XRD.

### 2.2. Catalytic Activity of the Series of Heteropoly Compounds

The catalytic properties of the prepared series of catalysts in which the cobalt and iron were tested as countercation of the Keggin 12-heteropolytungstate (Co_1.5_PW_12_O_40_, Fe_1.5_PW_12_O_40_) and 12-heteropolymolybdate (Co_1.5_PMo_12_O_40_, and Fe_1.5_PMo_12_O_40_) were investigated. The conversion of methanol (*X*_c_) and the selectivity of the products are listed in Table 2. The results show that the conversion and the DMC selectivity obtained over the Fe_1.5_PMo_12_O_40_ catalyst were 24.18% and 0.18%, respectively. When the iron cation was replaced by cobalt cation, *X*_c_ raised to 0.51% and the DMC selectivity to 54.12%.

When the molybdenum addenda atom of the Fe_1.5_PMo_12_O_40_ catalyst was substituted by tungsten addenda atom, the conversion (0.51%) remained stable, whereas a remarkable increase in DMC selectivity (61.87%) was observed. Finally, when the iron cation of the tungstate metal salt was replaced by the cobalt cation, only a slight increase in the DMC selectivity (69.00%) was observed, whereas a remarkable increase in *X*_c_ (1.53%) was observed. It can be seen from these results that the conversion and the selectivity of DMC showed dependence on the type of metal constituting the counteraction and the polyanions. Co_1.5_PW_12_O_40_ was the most active and the most selective for the DMC formation of the series of catalysts, probably due to the remarkable synergistic effect between Co and W. Taking into account the above results, we can conclude that designing a catalyst system constituting of Co and W is suitable for the conversion of methanol and CO_2_ into dimethyl carbonate.

### 2.3. Catalytic Activity of Co_1.5_PW_12_O_40_

#### 2.3.1. Effect of Reaction Time

**Figure 2.**
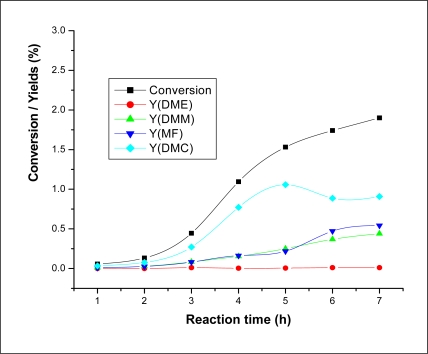
Conversion and DMC yield *versus* reaction time. Reaction conditions: m (catalyst) = 0.1 g; P_CO2_ = 2.5 bar.

Since the 12- tungstphosphate cobalt salt was found to be the best catalyst of the prepared series, it was chosen as the catalyst for the further study. In order to examine the variation of the conversion and the products formation during the reaction time, the reaction was carried out for 7 h at 80 °C and 2.5 bar using 0.1 g of Co_1.5_PW_12_O_40_ catalyst. Figure 2 shows the variation of the conversion and the yields as a function of reaction time. From this figure, it can be seen that the conversion and the yield for DMC production increased significantly in the initial 4 h. In fact, the conversion increased from 0.06% to 1.53% when the time increased from 1 h to 5 h. This increase corresponds to 96.08%. For further reaction time the conversion increased slightly. In fact, the conversion increased from 1.53% to 1.90% when the reaction time increased from 5 h to 7 h, which corresponds to an increase of 19.47%. As for the yield of DMC, it can be seen that in the first five hours, an increase of 96.23% was observed. Longer reaction times result in a formal decrease at about 6 h, and then the DMC yield remains stable. With longer reaction times (after 6 h), the continuous increase of the conversion along with the yield of dimethoxy methane (DMM) and methyl formate (MF), while a decrease of that of DMC was observed means that the system appears to approach an equilibrium state between DMC production and DMC hydrolysis. A kinetic study of the DMC decomposition is required to explain the decrease of the DMC yield in favor of DMM and MF.

**Figure 3.**
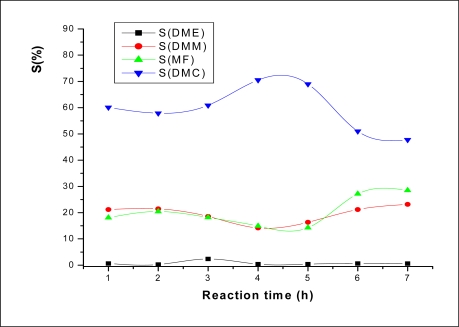
The effect of reaction time on product selectivity. Reaction conditions: m (catalyst) = 0.1 g; P_CO2_ = 2.5 bar.

As for the selectivities of the products (Figure 3), the change trend of the DMC selectivity was the same as that of DMC yield. The maximum of the DMC selectivity observed at 5 h was to the detriment to that of DMM and MF.

#### 2.3.2. Effect of the Support

**Figure 4.**
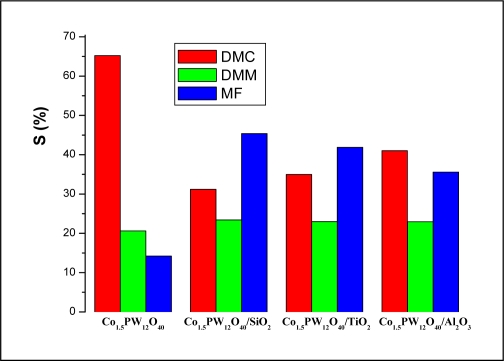
The effect of the support on the product selectivity. Reaction conditions: catalytic mass = 0.1 g; reaction temperature 80 °C; pressure of CO_2_ = 5 bar.

The activity of Co_1.5_PW_12_O_40_ in the synthesis of DMC from methanol and CO_2_ on the different supports, Al_2_O_3_ SiO_2_ and TiO_2_, was examined at 80 °C and at a pressure of CO_2_ equal to 5 bar. The results are summarized in Table 3. From these results, it can be concluded that the support significantly affects the conversion. When Al_2_O_3_ was used as a support, the activity (mol%/g of HPC) of the Co_1.5_PW_12_O_40_ changed from 3.73% (for the unsupported) to 39.33% (for the Al_2_O_3_-supported). Relatively high methanol conversion activity was obtained when the Al_2_O_3_ support was used. As for the products distribution (Figure 4), it can be seen that the DMC selectivity decreased when the Co_1.5_PW_12_O_40_ was supported on the kind of supports characterized as weakly basic. The decrease of DMC selectivity was in favor of that of MF, while the DMM selectivity remained almost unchanged (≈23%). The SiO_2_ and TiO_2_ (≈42–45%) support formed more MF than Al_2_O_3_ (≈36%). This indicates that the properties of the supports can influence the performance of the catalysts considerably. According to Ref. [29], the formation of MF on supported heteropoly compound catalysts requires the presence of both isolated oxo-metal sites and methoxy groups on the support, which can be formed from the interaction of methanol with hydroxyl groups of titania. Damyanova *et al.* [30] mentioned that the free titania surface could induce an increased methoxy group concentration and hence the formation of MF. This hints that a weak basic character of the support is more advantageous for this reaction. The enhanced *X*_c_ and DMC selectivity was probably due to the fact that Al_2_O_3_ is less acidic than SiO_2_ and TiO_2_. In fact, it was reported that the acidity of Al_2_O_3_ support can be increased by the addition of SiO_2_ and TiO_2_ in the preparation of Al_2_O_3_ support [31,32].

## 3. Experimental Section

### 3.1. Catalyst Preparation

The heteropolytungstate (Co_1.5_PW_12_O_40_; Fe_1.5_PW_12_O_40_) and the heteropolymolybdate transition metal salts (Co_1.5_PMo_12_O_40_; Fe_1.5_PW_12_O_40_) were prepared from 12-tungstophosphoric acid H_3_PW_12_O_40_, and 12-molybdophosphoric acid H_3_PMo_12_O_40_, respectively. These two heteropolyacids were prepared according to the methods of Deltcheff *et al.* [33]. The salt forms were obtained from their counterpart heteropolyacids, as precipitate by adding slowly the required amount of Ba(OH)_2_.8H_2_O (to neutralize the three protons) to the aqueous solution of the heteropolyacid, and then the required amount of MSO_4_.*x*H_2_O was added (M = Co; Fe). After eliminating the formed BaSO_4_ precipitate, the obtained solution was allowed to stand for few days at 4 °C. The salt was recovered from the solution by filtration. The series of supported catalysts (Co_1.5_PW_12_O_40_/Al_2_O_3_, Co_1.5_PW_12_O_40_/SiO_2_ and Co_1.5_PW_12_O_40_/TiO_2_) with a composition HPC/support = 30/70 was prepared by incipient-wetness impregnation. The support (Al_2_O_3_, SiO_2_, or TiO_2_) was impregnated with aqueous solutions of Co_1.5_PW_12_O_40_ with concentrations high enough to avoid its degradation [34]. The slurry of support and impregnation solution was constantly stirred at 50 °C until dryness evaporation. The catalyst was then dried over night at 120 °C.

### 3.2. Physicochemical Techniques

The purity and the Keggin structure of the samples were characterized by means of IR and XRD. IR spectra were recorded with an infrared spectrometer GENESIS II-FT-IR (4000−400 cm^−1^) as KBr pellets. The XRD powder patterns were recorded on a Rigaku diffractometer Ultima IV using CuKα radiation.

### 3.3. Reaction Procedure

Catalytic performance was tested in a stainless steel 250 mL autoclave equipped with a magnetic stirrer. The temperature of the autoclave was adjusted by a heating jacket. In a typical procedure, 20 mL of methanol and 0.1 g of catalyst were charged into the autoclave. CO_2_ was injected in to a low pressure, and then released, which was repeated two or three times in order to remove the air from the reactor. Following this, CO_2_ was injected to 2.5 bars. The system was stirred and heated at 80 °C for 5 h. After the reaction, the reactor was cooled down to less than 5 °C with a circulator and depressurized. The resulting solution was analyzed with a gas phase chromatograph (Agilent 6890N) equipped with a flame ionization detector, a thermal conductivity detector and a capillary column (HP-PLOT Q length 30 m ID 0.53 mm).

## 4. Conclusion

The catalytic properties of the prepared 12-heteropolytungstates (Co_1.5_PW_12_O_40_, Fe_1.5_PW_12_O_40_) and 12-heteropolymolybdates (Co_1.5_PMo_12_O_40_, and Fe_1.5_PMo_12_O_40_) were investigated. It was found that Co_1.5_PW_12_O_40_ was the most active and selective catalyst for the direct synthesis of DMC from methanol and CO_2_. Its high catalytic activity can be attributed to the synergetic effect between Co and Fe.

The methanol conversion activity was drastically increased whereas the DMC selectivity was decreased if Co_1.5_PW_12_O_40_ was supported on an acidic or a weak basic support. Thus, the direct conversion of the methanol in DMC can be enhanced if the 12-tungstophosphate cobalt salt is supported on a support that is adequately basic.

## Figures and Tables

**Table 1.** The relevant frequencies (cm^−1^) of PW_12_O_40_ and PMo_12_O_40_ having cobalt and iron as countercations.

**Catalyst**	**Frequency (cm^−1^)**			
	**ν_as_ (P-O_a_)**	**ν_as_ (M-O_d_)**	**ν_as_ (M-O_b_-M)**	**ν_as_(M-O_c_-M)**

Fe_1.5_PMo_12_O_40_	1064.71	960.55	867.97	783.10
Co_1.5_PMo_12_O_40_	1062.78	960.55	871.82	785.03
Fe_1.5_PW_12_O_40_	1080.14	981.77	894.97	806.25
Co_1.5_PW_12_O_40_	1080.14	979.84	894.97	790.00

**Table 2.** Conversion of methanol (*X*_c_) and product selectivities obtained from the reaction of methanol with CO_2_. Reaction conditions: catalytic mass = 0.1 g; reaction temperature 80 °C; pressure of CO_2_ = 2.5 bar.

**Catalyst *X*_c_ (%)**	**S (%)**				
		**DME**	**DMM**	**MF**	**DMC**

Fe_1.5_P Mo_12_O_40_	0.18	2.00	27.99	45.84	24.18
Co_1.5_PMo_12_O_40_	0.51	0.70	16.11	29.07	54.12
Fe_1.5_PW_12_O_40_	0.51	0.10	24.43	13.60	61.87
Co_1.5_PW_12_O_40_	1.53	0.38	16.35	14.27	69.00

**Table 3.** The effect of the support on the conversion and DMC yield. Reaction conditions: catalytic mass = 0.1 g; reaction temperature 80 °C; pressure of CO_2_ = 5 bar.

**Catalyst (mol%/1g-HPC)**	**Conv (%)**	**Conv (%)**	**Yield (%)**		
			**DMM**	**MF**	**DMC**

Co_1.5_PW_12_O_40_	3.73	3.73	0.77	0.53	2.43
Co_1.5_PW_12_O_40_/SiO_2_	1.12	37.33	0.26	0.51	0.35
Co_1.5_PW_12_O_40_/TiO_2_	0.89	29.67	0.21	0.37	0.31
Co_1.5_PW_12_O_40_/Al_2_O_3_	1.18	39.33	0.27	0.42	0.48
